# Regulating Charge and Exciton Distribution in High-Performance Hybrid White Organic Light-Emitting Diodes with n-Type Interlayer Switch

**DOI:** 10.1007/s40820-017-0138-4

**Published:** 2017-03-17

**Authors:** Dongxiang Luo, Yanfeng Yang, Ye Xiao, Yu Zhao, Yibin Yang, Baiquan Liu

**Affiliations:** 1 School of Materials and Energy, Guangdong University of Technology, Guangzhou, 510006 People’s Republic of China; 2 Institute of Polymer Optoelectronic Materials and Devices, State Key Laboratory of Luminescent Materials and Devices, South China University of Technology, Guangzhou, 510640 People’s Republic of China; 3 LUMINOUS! Center of Excellence for Semiconductor Lighting and Displays, School of Electrical and Electronic Engineering, Nanyang Technological University, Nanyang Avenue, Singapore, 639798 Singapore

**Keywords:** White light, Hybrid, Interlayer, Color rendering index, Organic light-emitting diodes

## Abstract

The interlayer (IL) plays a vital role in hybrid white organic light-emitting diodes (WOLEDs); however, only a negligible amount of attention has been given to n-type ILs. Herein, the n-type IL, for the first time, has been demonstrated to achieve a high efficiency, high color rendering index (CRI), and low voltage trade-off. The device exhibits a maximum total efficiency of 41.5 lm W^−1^, the highest among hybrid WOLEDs with n-type ILs. In addition, high CRIs (80–88) at practical luminances (≥1000 cd m^−2^) have been obtained, satisfying the demand for indoor lighting. Remarkably, a CRI of 88 is the highest among hybrid WOLEDs. Moreover, the device exhibits low voltages, with a turn-on voltage of only 2.5 V (>1 cd m^−2^), which is the lowest among hybrid WOLEDs. The intrinsic working mechanism of the device has also been explored; in particular, the role of n-type ILs in regulating the distribution of charges and excitons has been unveiled. The findings demonstrate that the introduction of n-type ILs is effective in developing high-performance hybrid WOLEDs. 
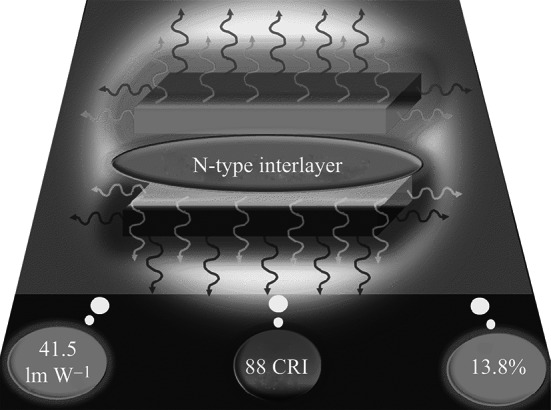

## Highlights

- The n-type interlayer was demonstrated to achieve a high efficiency, high color rendering index (CRI), and low voltage trade-off. The device exhibits a maximum total efficiency of 41.5 lm W^−1^ and a low turn-on voltage of 2.5 V (>1 cd m^−2^).
- High CRIs (80–88) at practical luminances (≥1000 cd m^−2^) were obtained, with a CRI of 88 being the highest among hybrid WOLEDs.

## Introduction

White organic light-emitting diodes (WOLEDs) have been aggressively explored for display and solid-state lighting applications because of their excellent characteristics such as high efficiency, low power consumption, fast switching, and flexibility [1–5]. In general, there are three types of WOLEDs depending on the employed emissive material, which are all-phosphorescent, all-fluorescent, and hybrid WOLEDs. Among WOLEDs, the utilization of phosphorescent (P) emitters is desirable as phosphors can allow for an up to 100% efficiency in converting injected charges into emitted photons (both singlet and triplet excitons are harvested), resulting in a theoretical internal quantum efficiency of unity [6–10]. Unfortunately, until now, no appropriate blue P material could be obtained in terms of lifetime and color stability, restricting the development of all-phosphorescent WOLEDs.

To solve the above issue, researchers have devoted their attention to pursuing hybrid WOLEDs, which combine fluorescent (F) blue emitters with P green–red/orange emitters to generate white emission as blue F emitters can exhibit long lifetimes and stable colors [11, 12]. To date, two approaches had been used to create hybrid WOLEDs according to the triplet energy (T_1_) of blue F emitters. One is composed of blue fluorophores with triplet energies, which are higher than that of complementary phosphors [13–19]. Although this approach can simplify the structures, it is still difficult to synthesize blue F emitters with a high T_1_ [20, 21]. In addition, the lifetime of this type of hybrid WOLED is unsatisfactory. Conversely, hybrid WOLEDs can be fundamental to blue fluorophores with triplet energies, which are lower than that of complementary phosphors. Previously, the latter kind of hybrid WOLED had been demonstrated to have the potential to possess many merits, including high efficiency, low-efficiency roll-off, low voltage, stable color, high color rendering index (CRI), and long lifetime [11, 22–34]. Therefore, it is beneficial to further enhance the performance of the latter kind of hybrid WOLED because of their peculiar advantages.

A crucial feature of preparing the latter kind of hybrid WOLED is the use of an interlayer (IL), located between the blue F emitter and complementary P emitter, which can (1) prevent the Förster energy transfer from F blue emitters to red–green/orange P emitters, (2) eliminate the Dexter energy transfer from red–green/orange P emitters to F blue emitters, (3) tune the emission colors, and (4) prolong the lifetime [11, 22–34]. In fact, a large number of materials have been used as effective ILs to realize hybrid WOLEDs, such as the most widely used 4,4-*N*,*N*-dicarbazolebiphenyl (CBP) [11, 22–24], 4,4′,4″-tri(9-carbazoyl) triphenylamine (TCTA): 2,2′,2″-(1,3,5-benzinetriyl)-tris(1-phenyl-1-*H*-benzimidazole) (TPBi) used by Leo group [25, 26], and bis[2-(2-hydroxyphenyl)-pyridine] beryllium (Bepp_2_) (TCTA) [27, 28], *N*,*N*′-di(naphthalene-1-yl)-*N*,*N*′-diphenyl-benzidine (NPB) [29, 30] and 1-bis[4-[*N*,*N*-di(4-tolyl)amino]phenyl]-cyclohexane (TAPC) used by Ma group [31]. It is noted that most publications are focused on bipolar or p-type ILs; however, only a negligible amount of attention has been given to n-type ILs. As a result, the performance (i.e., efficiency, CRI, and driving voltage) of hybrid WOLEDs with n-type ILs lags far behind their counterparts [32–34]. For instance, maximum power efficiencies (PEs) of only 18.8, 3.0, and 20.9 lm W^−1^ have been obtained in Ho’s [32], Xia’s [33], and our [34] hybrid WOLEDs with the n-type IL, respectively. In addition, the introduction of n-type ILs to regulate charges and excitons, which helps to boost performance, is not well understood. Moreover, the comparison of bipolar, p-type, and n-type ILs, which can be very helpful in understanding the role of ILs in hybrid WOLEDs, is not clear. Therefore, is it possible to further enhance the performance of hybrid WOLEDs with n-type ILs?

In this paper, the hybrid WOLED based on n-type ILs, for the first time, has been demonstrated to achieve a high efficiency, high CRI, and low voltage trade-off. The optimized device exhibits a maximum total efficiency of 41.5 lm W^−1^, the highest value among hybrid WOLEDs with n-type ILs. In addition, high CRIs (80–88) at practical luminances (≥1000 cd m^−2^) have been obtained, satisfying the demand for indoor lighting. Remarkably, a CRI of 88 is the highest among hybrid WOLEDs. Moreover, the device exhibits low voltages, with a turn-on voltage of only 2.5 V (>1 cd m^−2^), which is the lowest among hybrid WOLEDs. The intrinsic working mechanism of the device has also been explored; in particular, the role of n-type ILs in regulating the distribution of charges and excitons has been unveiled. The findings demonstrate that the introduction of n-type ILs is effective in developing high-performance hybrid WOLEDs.

## Experimental

**Fig. 1:**
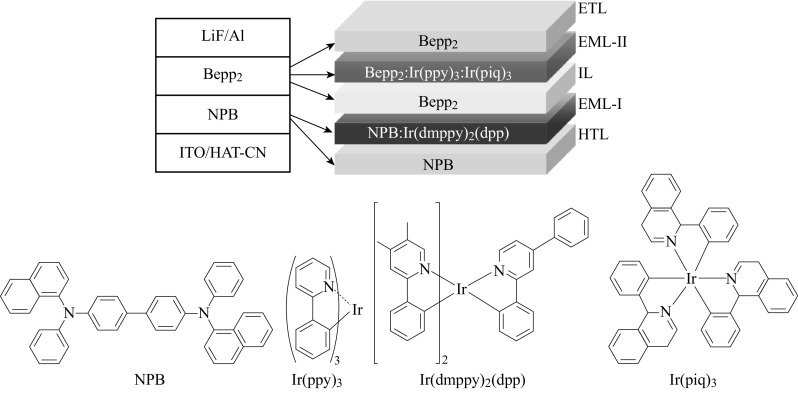
*Top* schematic layer structure of the WOLED. *Bottom* chemical structure of emitters

As depicted in Fig. 1, the configuration of the hybrid WOLED with n-type ILs (device W1) is ITO/HAT-CN (100 nm)/NPB (20 nm)/NPB: Ir(dmppy)_2_(dpp) (20 nm, 1.5%)/Bepp_2_ (3.5 nm)/Bepp_2_: Ir(ppy)_3_: Ir(piq)_3_ (15 nm, 1: 6%: 1.3%)/Bepp_2_ (35 nm)/LiF (1 nm)/Al (200 nm). ITO is indium tin oxide (the anode), HAT-CN is 1,4,5,8,9,11-hexaazatriphenylene hexacarbonitrile [the hole injection layer], the 20-nm NPB functions as the hole transport layer (HTL), bis(2-phenyl-4,5-dimethylpyridinato)[2-(biphenyl-3-yl)pyridinato] iridium(III) (Ir(dmppy)_2_(dpp), a yellow emitter) was doped into the NPB host as emitting layer-I (EML-I), the 3.5-nm Bepp_2_ acts as the n-type IL, tris(2-phenylpyridinne)iridium(III) [Ir(ppy)_3_] and tris(1-phenylisoquinolinolato-*C* ^2^,*N*) iridium(III) [Ir(piq)_3_] were co-doped into the Bepp_2_ host as the EML-II, the 35-nm Bepp_2_ acts as the electron transport layer (ETL) because of its high electron mobility of 10^−4^ cm^2^ (Vs)^−1^ [28], LiF is the electron injection layer, and Al is the cathode. The detailed fabrication and measurement of the devices follow well-established processes, as reported in another paper [31].

## Discussion and Results

**Fig. 2:**
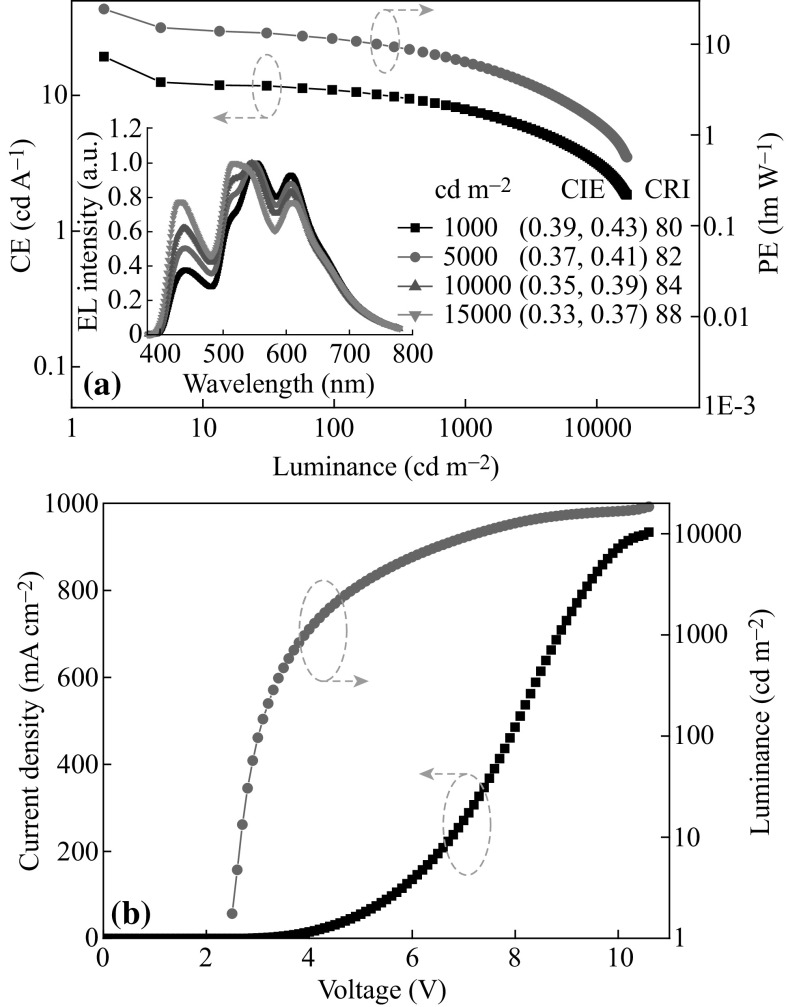
**a** Forward-viewing current and power efficiencies as a function of luminance. *Inset* EL spectra of W1 at various luminances (CIE is the Commission International de l’Eclairage coordinates). **b** Current density–voltage–luminance curves of W1

The CE and PE of device W1, which depends on the luminance, are clearly shown in Fig. 2. A maximum forward-viewing CE and PE of 19.4 cd A^−1^ and 24.4 lm W^−1^ are obtained, respectively. As illumination sources are typically characterized by their total emitted power [11], the maximum total PE is 41.5 lm W^−1^, which is the highest value among hybrid WOLEDs with n-type ILs. In fact, the efficiency (41.5 lm W^−1^) is also higher than that of recent hybrid WOLEDs [35–42], indicating that n-type ILs are effective in achieving high-efficiency WOLEDs. The maximum total external quantum efficiency (EQE) of W1 is 13.8%. In addition, as displayed in the inset in Fig. 2a, W1 exhibits high CRIs (80–88) at practical luminances (≥1000 cd m^−2^), indicating that W1 can satisfy the demand for indoor lighting [3]. Remarkably, a CRI of 88 is among the highest in hybrid WOLEDs. Moreover, W1 exhibits very low voltages, as shown in Fig. 2b. For example, the turn-on voltage is only 2.5 V (for a luminance of >1 cd m^−2^), which is the lowest among hybrid WOLEDs. At 100 and 1000 cd m^−2^, the voltages are 3.0 and 3.95 V, respectively. As it is still a challenge for WOLEDs to achieve low driving voltages for practical use (e.g., <3 V for onset and <4 V at 100 cd m^−2^ for portable displays) [43], it is clear that our device can effectively alleviate this difficulty. It is important to note that a much higher efficiency and lower voltage can be expected if a p-i-n structure is used. In brief, W1 successfully achieves a high efficiency, high CRI, and low voltage trade-off, which cannot be realized by previous hybrid WOLEDs with n-type ILs.

Motivated by this excellent performance, we subsequently performed a detailed study on the intrinsic working mechanism of W1, which will make it easier to understand hybrid WOLEDs with n-type ILs, in particular the role of n-type ILs in regulating the distribution of charges and excitons.

**Fig. 3:**
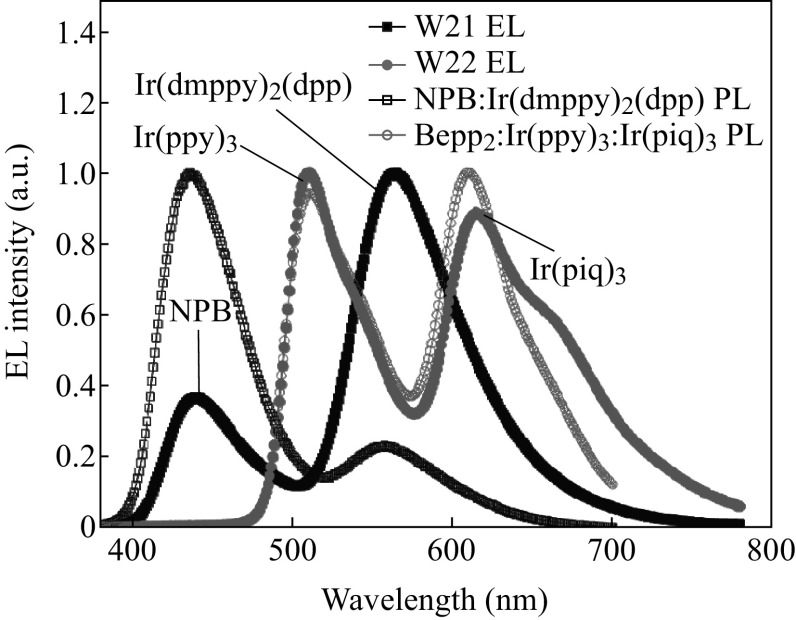
EL spectra of W21 and W22 at 1000 cd m^−2^, PL spectra of the NPB: Ir(dmppy)_2_(dpp) (30 nm, 1.5%) film and the Bepp_2_: Ir(ppy)_3_: Ir(piq)_3_ (30 nm, 1: 6%: 1.3%) film

First, four emitters have been used to generate white emission, guaranteeing high CRIs. By using the double EMLs, four clear emission peaks can be observed, where the peaks of approximately 440, 510, 560, and 620 nm originate from NPB, Ir(ppy)_3_, Ir(dmppy)_2_(dpp), and Ir(piq)_3_, respectively. More specifically, the blue and yellow emissions are generated from NPB and Ir(dmppy)_2_(dpp) in EML-I, respectively, while the green and red emissions are from Ir(ppy)_3_ and Ir(piq)_3_ in EML-II, respectively. To verify the above analyses, two devices with a single EML corresponding to each EML of W1 have been fabricated, where the structures are ITO/HAT-CN (100 nm)/NPB (20 nm)/NPB: Ir(dmppy)_2_(dpp) (20 nm, 1.5%)/Bepp_2_ (35 nm)/LiF (1 nm)/Al (200 nm) and ITO/HAT-CN (100 nm)/NPB (15 nm)/TCTA (5 nm)/Bepp_2_: Ir(ppy)_3_: Ir(piq)_3_ (20 nm, 1: 6%: 1.3%)/Bepp_2_ (35 nm)/LiF (1 nm)/Al (200 nm) for the EML-I-based device (W21) and the EML-II-based device (W22), respectively. As shown in Fig. 3, the NPB and Ir(dmppy)_2_(dpp) emissions are clearly observed in W21, while the Ir(ppy)_3_ and Ir(piq)_3_ emissions can be clearly observed in W22, indicating that the white emissions originate from NPB, Ir(ppy)_3_, Ir(dmppy)_2_(dpp), and Ir(piq)_3_. To further understand the energy transfer properties from the hosts to the dopant phosphors, the photoluminescent (PL) characteristics of the doping films based on the NPB and Bepp_2_ host and corresponding dopants have been measured, as shown in Fig. 3. For the NPB: Ir(dmppy)_2_(dpp) film, the PL emission peaks from NPB and Ir(dmppy)_2_(dpp) can be clearly observed, indicating that the energy transfer from NPB to Ir(dmppy)_2_(dpp) is incomplete. For the Bepp_2_: Ir(ppy)_3_: Ir(piq)_3_ film, only the PL emission peaks from Ir(ppy)_3_ and Ir(piq)_3_ can be observed, with no Bepp_2_ emission visible, indicating that the energy transfer from Bepp_2_ to the dopants is efficient. Hence, the above facts further demonstrate that the white emissions originate from the NPB, Ir(ppy)_3_, Ir(dmppy)_2_(dpp), and Ir(piq)_3_.

As the generation of blue emission is the essential device for engineering hybrid WOLEDs, the role of n-type ILs in regulating the distribution of charges and excitons can be revealed by analyzing the blue emission intensities [4, 44]. For the blue emission of W1, although the T_1_ of NPB (2.3 eV) is higher than that of Ir(dmppy)_2_(dpp) (<2.25 eV) [4], the energy transfer from the NPB host to the Ir(dmppy)_2_(dpp) guest in EML-I is incomplete because of the relatively low Ir(dmppy)_2_(dpp) concentration of 1.5% (as demonstrated above), leading to the fact that the NPB host can generate blue fluorescence emission [14]. In addition, by using NPB as the emitter, (1) the heterojunction that exists between the HTL and EML-I is eliminated, which is beneficial to the low voltage and long lifetime [45]; (2) the number of evaporation sources used in the fabrication process is reduced as there is no need to prepare another source for the HTL. To ensure that enough excitons can be harvested for the strong blue intensity and high performance, the n-type IL is essential. Before demonstrating the importance of the n-type IL, the main exciton generation zone of W1 was clarified.

**Fig. 4:**
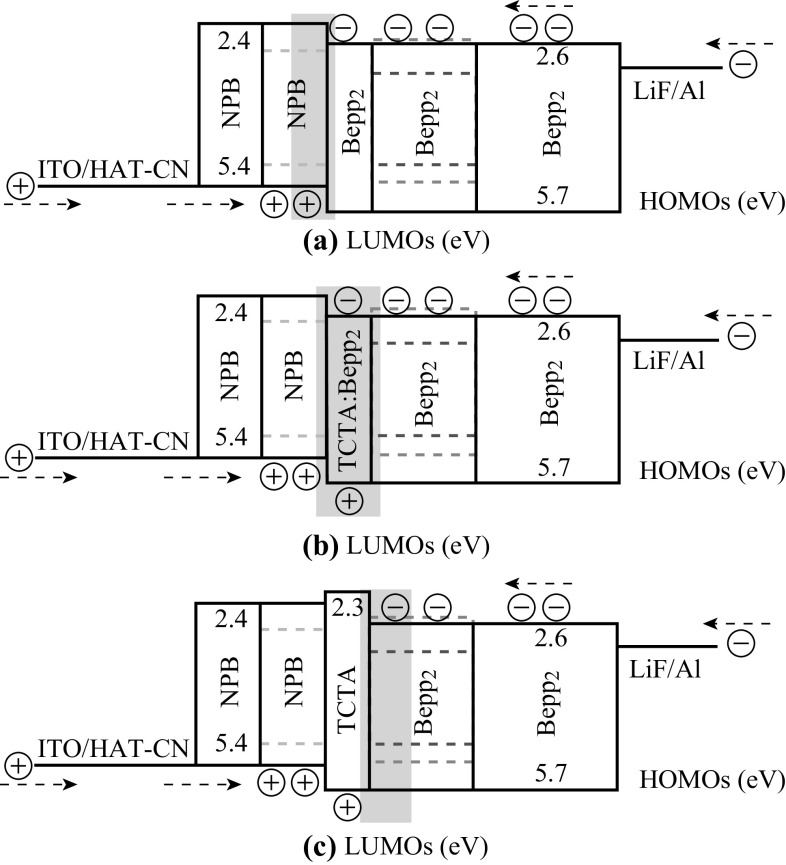
Schematic of the distribution of charges and excitons of **a** W1 with n-type IL, **b** W3 with bipolar IL, and **c** W4 with p-type IL. The gray-filled rectangles are the main exciton generation zones

As Bepp_2_ is an n-type material, electrons injected from the cathode can be feasibly transported to the NPB/Bepp_2_ interface although a small part of them may be trapped by Ir(ppy)_3_ and Ir(piq)_3_. Meanwhile, holes injected from the anode can easily arrive at the NPB/Bepp_2_ interface because of the strong hole injecting/transporting ability of HAT-CN/NPB [4]. As a result, the main exciton generation zone of W1 is located at the NPB/Bepp_2_ interface, resulting in the formation of both singlet and triplet excitons at this interface with a ratio of 1:3 [1], as shown in Fig. 4a. However, it should be noted that some holes can reach the EML-II region where the electrons are located as (1) the highest occupied molecular orbital (HOMO) barrier between NPB and Bepp_2_ is not too high (only 0.3 eV), indicating that holes may overcome this barrier upon obtaining enough energy; (2) Bepp_2_ may not totally block the transport of holes considering that excitons can be formed for the device using a >20-nm Bepp_2_ as the EML/hole blocking layer [41]. As a result, some of the excitons can be directly generated on the Bepp_2_ host or guests, increasing the green and red emissions, although this is not the main exciton generation zone. Therefore, without effective ILs, the triplets generated in EML-II can be quenched by NPB as the T_1_ of NPB is lower than that of Ir(ppy)_3_ (2.4 eV) and Bepp_2_ (2.6 eV) [11, 28], leading to a low efficiency. The Bepp_2_ IL indicates that the main exciton generation zone of W1 is located at the NPB/Bepp_2_ interface, guaranteeing that a sufficient number of singlets can be harvested by NPB to generate blue emission; otherwise, only a poor blue intensity can be produced. However, it should be noted that the efficiency roll-off problem can occur at the NPB/Bepp_2_ interface, which may be alleviated if bipolar blue emitters are used because bipolar materials can broaden the excitation zone.

**Fig. 5:**
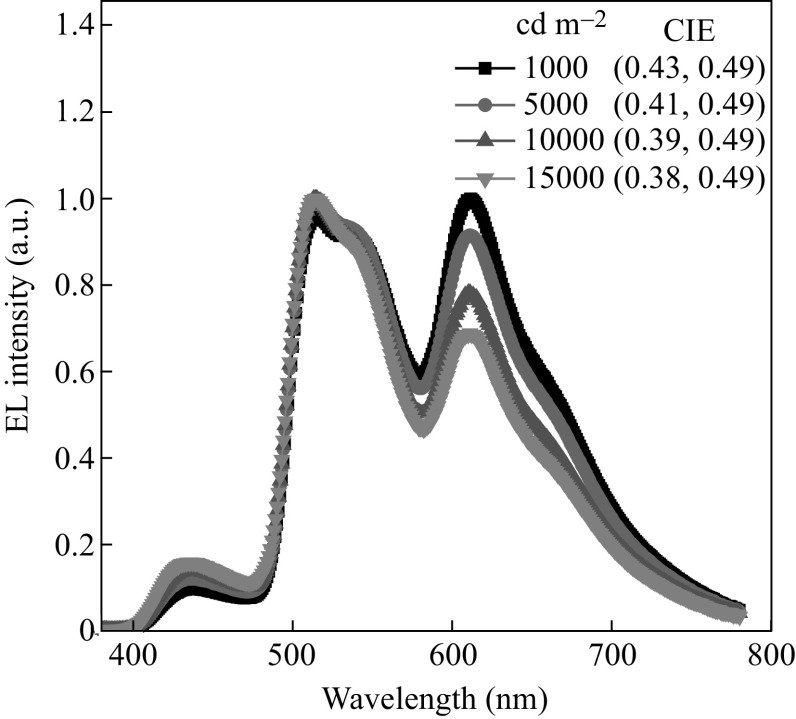
EL spectra of W3 at various luminances

To further verify the significance of the Bepp_2_ IL, a device (W3) with the bipolar IL, by co-doping TCTA and Bepp_2_ (with a ratio of 1:1, 3.5 nm), has been fabricated, while the other layers are similar to those of W1 except for the IL. TCTA is selected because its hole mobility is almost identical to the electron mobility of Bepp_2_ [28], which can effectively balance the charge transport in the IL. By inserting the bipolar IL, the main exciton generation zone of W3 is different from that of W1, as shown in Fig. 4b. As TCTA is a p-type material, holes are more easily transported to EML-II, whereas it is difficult for electrons to arrive at EML-I in W3 compared with W1, leading to the formation of additional excitons in EML-II but less in EML-I. Thus, it is reasonable that strong green/red emissions but poor blue emission are observed in W3, as shown in Fig. 5. Although the main exciton generation zone of W3 is relatively wide, the maximum CRI of W3 is only 82 because of the poor blue intensity, lower than that of W1, indicating that the n-type IL is more effective than the bipolar IL in guaranteeing high CRIs in our device, which is unlike previous reports [27, 28].

**Fig. 6:**
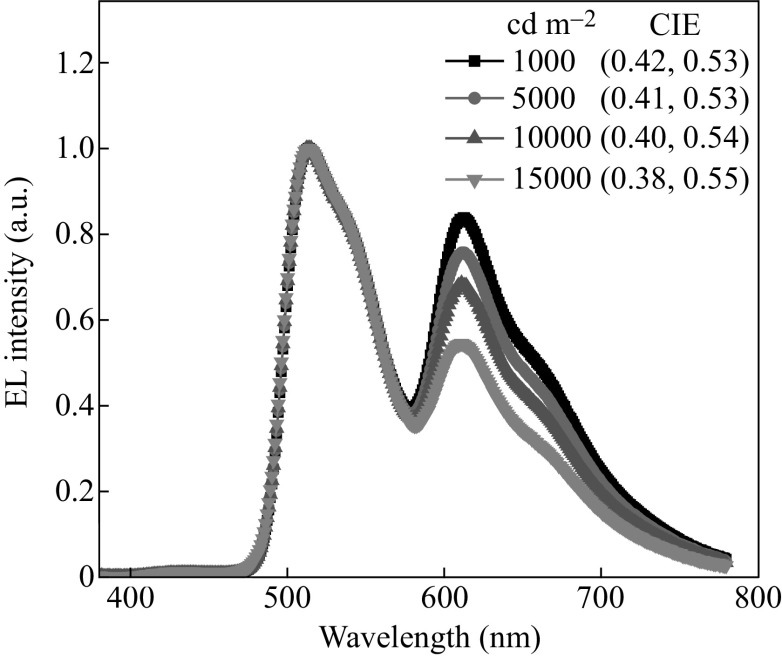
EL spectra of W4 at various luminances

To further understand the distribution of charges and excitons, which can be regulated by the IL switch, as well as systematically compare the effect of different kinds of ILs, a device (W4) with a 3.5-nm TCTA as the p-type IL was fabricated, while the other layers are similar to those of W1 except for the IL. Because an energy barrier exists between TCTA and Bepp_2_ together with the fact that TCTA and Bepp_2_ are p-type and n-type materials, respectively, the main exciton generation zone of W4 is located at the TCTA/Bepp_2_ interface, as shown in Fig. 4c. For W4, only a small amount of electrons can pass through the 3.5-nm TCTA to reach EML-I because of the higher lowest unoccupied molecular orbital (LUMO) and very weak electron mobility of TCTA (2.3 eV) [28]. As a result, fewer excitons can be formed in EML-I. Thus, even at a high luminance/voltage, almost no blue emission can be observed in W4, leading to no white emission, as shown in Fig. 6.

**Fig. 7:**
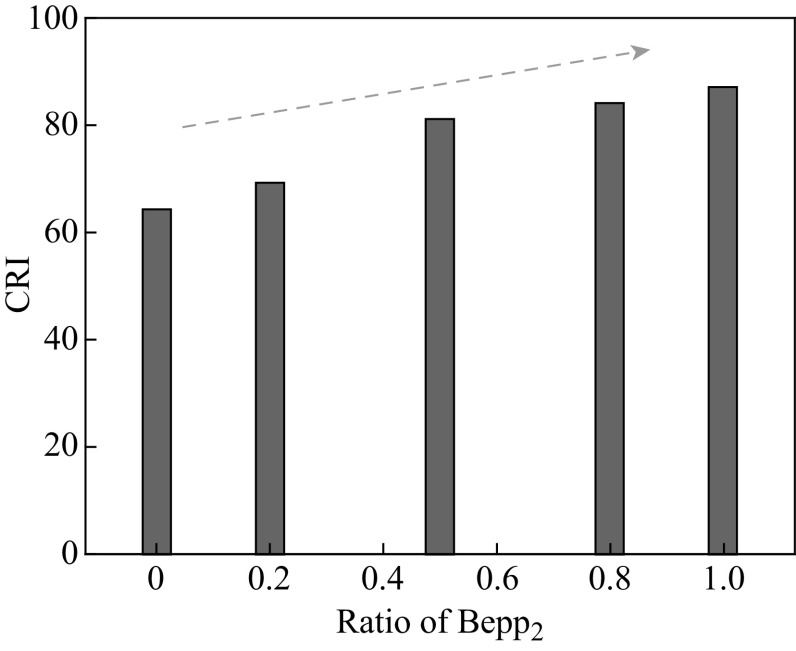
Maximum CRIs of devices as a function of the ratio of Bepp_2_ in TCTA: Bepp_2_ ILs

Moreover, to better illustrate the role of ILs in regulating the distribution of charges and excitons, the maximum CRI of devices using various ratios of TCTA and Bepp_2_ as the ILs was measured, as shown in Fig. 7. As a certain amount of blue emission is required to ensure a high CRI, excitons should be harvested by NPB as much as possible. The lowest CRI of 65 is obtained only when the TCTA IL is used as almost no excitons can be harvested by NPB to generate blue emission, as mentioned above. Conversely, the highest CRI of 88 is obtained only when Bepp_2_ is used as a sufficient number of excitons can be confined to EML-I to be harvested by NPB because of the n-type IL. As the ratio of Bepp_2_ increases, the CRI increases, indicating that the n-type IL is essential in guaranteeing a sufficient amount of blue emission for a high CRI.

Finally, it should be noted that the T_1_ of ILs has been demonstrated to be significant to hybrid WOLEDs [34]. In our devices, both Bepp_2_ and TCTA possess high T_1_ values (i.e., 2.6 and 2.8 eV for Bepp_2_ and TCTA, respectively) [44], which would not quench the generated triplets. As shown in Fig. 1, because Bepp_2_ functions as the IL, the host of EML-II and ETL in W1, only three organic layers exist, which is much less than those of previous multi-EML hybrid WOLEDs and even less than those of single-EML hybrid WOLEDs [14, 15]. Thus, the structure of W1 with the n-type IL is more simplified than that of W3 with the bipolar IL or W4 with the p-type IL. In addition, the heterojunction that exists between the IL and EML-II and the heterojunction that exists between EML-II and ETL are eliminated because of the multifunctional role of Bepp_2_, which cannot be realized by the bipolar or p-type IL-based devices. Moreover, the number of evaporation sources used in the fabrication process is reduced in W1 compared to W3 or W4 as there is no need to prepare another source for the IL. Therefore, the n-type IL, which was previously overlooked, has been demonstrated to possess many advantages in our device structures. In brief, the n-type IL can (1) regulate the distributions of charges and excitons, (2) simplify the device structure, (3) eliminate the heterojunctions, and (4) reduce the number of evaporation sources.

## Conclusions

We have demonstrated that the hybrid WOLED with n-type ILs can achieve a high efficiency, high CRI, and low voltage trade-off. The device can exhibit (1) an unprecedented efficiency of 41.5 lm W^−1^ for hybrid WOLEDs with n-type ILs, (2) high CRIs (80–88) at practical luminances (≥1000 cd m^−2^), and (3) low voltages (i.e., 2.5 V for >1 cd m^−2^). The intrinsic working mechanism of the device has also been explored. Particularly, the role of n-type ILs in regulating the distribution of charges and excitons has been unveiled. The findings demonstrate that the introduction of n-type ILs is effective in developing high-performance hybrid WOLEDs, which may guide the rational design of both the material and device structure of WOLEDs in emerging display and lighting applications.
